# *In Silico* Identification of Highly Conserved Epitopes of Influenza A H1N1, H2N2, H3N2, and H5N1 with Diagnostic and Vaccination Potential

**DOI:** 10.1155/2015/813047

**Published:** 2015-08-06

**Authors:** José Esteban Muñoz-Medina, Carlos Javier Sánchez-Vallejo, Alfonso Méndez-Tenorio, Irma Eloísa Monroy-Muñoz, Javier Angeles-Martínez, Andrea Santos Coy-Arechavaleta, Clara Esperanza Santacruz-Tinoco, Joaquín González-Ibarra, Yu-Mei Anguiano-Hernández, César Raúl González-Bonilla, Eva Ramón-Gallegos, José Alberto Díaz-Quiñonez

**Affiliations:** ^1^Laboratorio Central de Epidemiología, Centro Médico Nacional La Raza, Instituto Mexicano del Seguro Social, Avenida Jacarandas S/N, Esquina Circuito Interior, Colonia La Raza Delegación Azcapotzalco, 02990 México, DF, Mexico; ^2^Laboratorio de Biotecnología y Bioinformática Genómica, Escuela Nacional de Ciencias Biológicas, Instituto Politécnico Nacional, Carpio y Plan de Ayala S/N, Colonia Plutarco Elías Calles, Delegación Miguel Hidalgo, 11340 México, DF, Mexico; ^3^Laboratorio de Genómica, Departamento de Genética y Genómica Humana, Instituto Nacional de Perinatología “Isidro Espinosa de los Reyes”, Montes Urales 800, Colonia Lomas Virreyes, Delegación Miguel Hidalgo, 11000 México, DF, Mexico; ^4^Laboratorio de Genómica, Departamento de Biología Molecular, Instituto Nacional de Cardiología “Ignacio Chávez”, Juan Badiano No. 1, Colonia Sección XVI, Delegación Tlalpan, 14080 México, DF, Mexico; ^5^División de Laboratorios de Vigilancia e Investigación Epidemiológica, Instituto Mexicano del Seguro Social, Mier y Pesado No. 120, Colonia Del Valle, Delegación Benito Juárez, 03100 México, DF, Mexico; ^6^Laboratorio de Citopatologia Ambiental, Departamento de Morfología, Escuela Nacional de Ciencias Biológicas Campus Zacatenco, Instituto Politécnico Nacional, Wilfrido Massieu S/N, Colonia Nueva Industrial Vallejo, Delegación Gustavo A. Madero, 07738 México, DF, Mexico; ^7^Instituto de Diagnóstico y Referencia Epidemiológicos “Manuel Martínez Báez”, Francisco de P. Miranda No. 177, Colonia Unidad Lomas de Plateros, Delegación Álvaro Obregón, 01480 México, DF, Mexico; ^8^Facultad de Medicina, Universidad Nacional Autónoma de México, Avenida Universidad 3000, Colonia Copilco Universidad, Delegación Coyoacán, 04510 México, DF, Mexico

## Abstract

The unpredictable, evolutionary nature of the influenza A virus (IAV) is the primary problem when generating a vaccine and when designing diagnostic strategies; thus, it is necessary to determine the constant regions in viral proteins. In this study, we completed an *in silico* analysis of the reported epitopes of the 4 IAV proteins that are antigenically most significant (HA, NA, NP, and M2) in the 3 strains with the greatest world circulation in the last century (H1N1, H2N2, and H3N2) and in one of the main aviary subtypes responsible for zoonosis (H5N1). For this purpose, the HMMER program was used to align 3,016 epitopes reported in the Immune Epitope Database and Analysis Resource (IEDB) and distributed in 34,294 stored sequences in the Pfam database. Eighteen epitopes were identified: 8 in HA, 5 in NA, 3 in NP, and 2 in M2. These epitopes have remained constant since they were first identified (~91 years) and are present in strains that have circulated on 5 continents. These sites could be targets for vaccination design strategies based on epitopes and/or as markers in the implementation of diagnostic techniques.

## 1. Introduction

The influenza A virus (IAV) appears seasonally, causes annual epidemics, and occasionally presents a new strain with pandemic reach, leading to severe consequences for global health and for the global economy [1, 2]. Every year, influenza affects approximately 15% of the world population, which translates to 3 to 5 million infections and 500,000 deaths [3, 4].

IAV is an enveloped virus and a member of the orthomyxoviridae family; its genome consists of eight segments of simple chain RNA of negative polarity that code for 3 structural proteins (HA, NA, and M2), 1 membrane protein (M1), and 6 nonstructural proteins (NS1, NEP/NS2, PA, PB1, PB1-F2, and PB2) [5].

IAV is classified according to its two principal membrane antigens: HA (18 subtypes) and NA (11 subtypes) [6, 7]. In humans, 6 subtypes (H1, H2, H3, H5, H7, and H9) [8–10] have been detected; however, only 3 of those have crossed the species barrier and have the capacity to transmit from human to human (H1N1, H2N2, and H3N2) [11].

The following three subtypes are responsible for the pandemics of the past century: H1N1 (Spanish flu, 1918), H2N2 (Asian flu, 1957), and H3N2 (Hong Kong flu, 1968) [12].

The last influenza pandemic occurred in 2009 due to a new pandemic virus A H1N1 (A H1N1pdm 09). This virus was detected in 214 cities on 5 continents and, up to July 30, 2010, had caused 18,389 cases [4].

These pandemic viruses arose due to a combination of aviary, pig, and human influenza viruses [13–15] because, in contrast to other respiratory viruses, they present two mutation mechanisms: genetic and antigenic drift [16].

These mutation mechanisms confer an unpredictable, evolving character to the influenza viruses, which is the principal difficulty to overcome when designing a vaccine. This difficulty occurs because while vaccination has been an effective method against 60 to 90% of seasonal strains [17], it has not been effective against pandemic viruses [18].

Inactivated trivalent vaccines that contain the hemagglutinin (HA) protein of the influenza A (H1N1) and A (H3N2) viruses and influenza B virus are the only authorized commercial vaccines [19]. Due to the high mutation rate of HA, it is necessary to vaccinate every year, according to World Health Organization suggestions [20].

Antiviral strategies that are currently in development include neutralizing antibodies [21], small molecule inhibitors [22], and universal vaccines [23] against the influenza virus based on conserved epitopes. Universal vaccines are used as an alternative approach for improving immunogenicity and cross-protection against emerging strains, shortening production time, and reducing side effects [24–26].

Therefore, it is of vital importance to know the sites of the proteins or antigenic determinants among the different strains of the influenza virus that historically have been recognized by the immune system. Therefore, the present study completed an* in silico* identification of highly conserved epitopes with diagnostic and vaccination potential in the HA, NA, MP, and M2 proteins of the influenza virus that have been reported from 1918 to 2014 for the primary strains that have circulated in the world (H1N1, H2N2, H3N2, and H5N1).

## 2. Materials and Methods

### 2.1. *In Silico* Search and Attainment of Epitopes

To complete the epitope search on the IEDB site (http://www.iedb.org/), the following inclusion criteria were taken into account: experimentally reported epitopes for HA, NA, NP, and M2 proteins corresponding to the H1N1, H2N2, H3N2, and H5N1 subtypes of influenza A from 1918 up to 2011.

*Exclusion Criteria.* Epitopes corresponding to other influenza subtypes or proteins or that had not been verified experimentally were excluded.

*Elimination Criteria*. All sequences shorter than 7 amino acids or with erroneous information were eliminated; individual archives were created in FASTA format using the SeqBuilder program (DNASTAR Madison, Wisconsin, USA).

### 2.2. Sequence Download

Using the Pfam database (http://pfam.xfam.org/), sequences corresponding to the hemagglutinin (PF00509), neuraminidase (PF00064), nucleoprotein (PF00506), and matrix 2 (PF00599) protein families were downloaded from the Pfam-A entries along with sequences from the NCBI database, representing manually refined, high-quality families.

### 2.3. Construction of Hidden Markov Models and Epitope Alignments

Using the* hmmbuild* algorithm of the HMMER program (European Bioinformatics Institute), each one of the alignments downloaded from the Pfam database was used in the construction of hidden Markov models. This algorithm generates a file with the extension ^*∗*^.hmm that contains the consensus sequence for each family of proteins.

Subsequently, the* hmmalign* algorithm was used to align the epitopes with their corresponding hidden Markov models (^*∗*^.hmm). This algorithm generates a file with the extension ^*∗*^.sto that contains the epitopes aligned with the consensus sequence for each protein.

### 2.4. Epitope Selection

Based on the files generated by the* hmmalign* program, the regions that presented the greatest frequency of reports of epitopes, at least 2 standard deviations above the mean, were selected for each protein and strain analyzed. Subsequently, two new alignments were completed with the help of the* Megalign* program (DNASTAR Madison, Wisconsin, USA). The first alignment was between the present epitopes in each region to determine the consensus recognition sequence. The second was between this consensus sequence and some current strains that circulated between 2013 and 2014 to determine the similitude percentage with strains that currently circulate.

### 2.5. Protein Modeling

To complete the modeling of the consensus sequences generated using the* hmmbuild* program, mold structures downloaded from the Protein Data Bank (PDB) with similitude percentages ≥70% were utilized. With these structures as a template, a three-dimensional structure of each protein was modeled in the Swiss-Model (Swiss Institute of Bioinformatics Biozentrum, University of Basel, Switzerland) virtual platform; the consensus regions of each epitope were highlighted in the model utilizing the PyMOL program (Schrödinger K. K., Japan).

### 2.6. Phylogenetic Analysis

For each epitope group, a phylogenetic tree with the complete sequence of the protein to which it belongs was constructed using the MEGA program [27]; the tree was constructed with the Neighbor-Joining algorithm and with a bootstrap of 1000 replicas.

### 2.7. Strain Circulation Analysis

To determine the reach that using these epitopes would have, in terms of vaccination or diagnosis in the world population, an analysis of the strains containing the consensus epitopes determined in this study was completed; for each epitope, data from viruses isolated from cases that were reported were collected.

**Figure 1:**
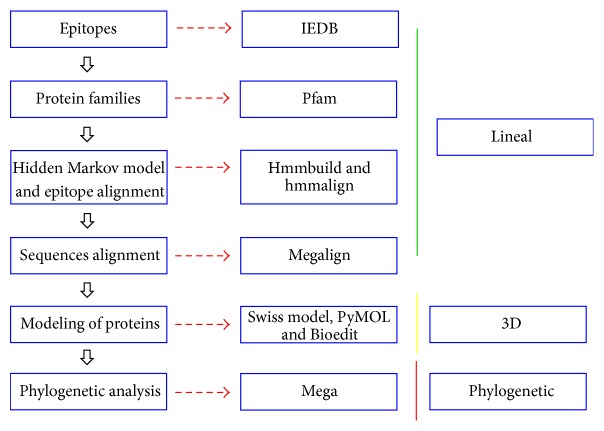
Flowchart of the stages and programs utilized in this study.

The stages of the entire analysis and the programs used are summarized in Figure 1.

### 2.8. Statistical Analysis

Descriptive statistics were used to determine the regions of the proteins that were recognized with the greatest frequency. For this purpose, the protein was divided into groups, each containing 10 amino acids, the frequencies of epitopes present in each group were observed, and the means and standard deviations were calculated. Up to 3 regions with frequencies at least 2 standard deviations above the mean were chosen for each protein.

## 3. Results

### 3.1. Distribution of Epitopes and Sequences

In total, 3,016 epitopes and 34,294 sequences were identified. Of the epitopes and sequences, respectively, 1,352 and 15,102 belonged to H1N1, 91 and 412 belonged to H2N2, 1,006 and 12,186 belonged to H3N2, and, finally, 567 and 6,594 belonged to H5N1. In all cases, HA is the protein for which more epitopes and sequences have been reported (Table 1).

### 3.2. Identification of the Region with the Greatest Frequency of Antigenic Recognition

**Figure 2:**
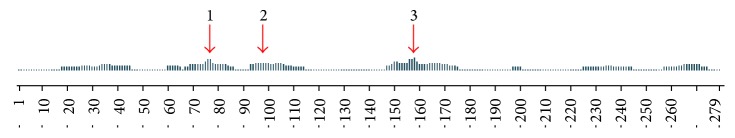
Example of the frequency distribution graph generated using the* hmmalign* program for hemagglutinin of the H2N2 subtype. The arrows indicate the selected regions for this protein.

Based on the frequency distribution graph generated using the* hmmalign* program, 25 sites were identified which showed the greatest frequency to which the epitopes aligned. These sites were distributed in the following manner: 8 for H1N1, 3 for H2N2, 6 for H3n2, and 8 for H5N1 (Table 2). All of these highly recognized regions had frequencies at least 3 standard deviations above the mean. An example of the graph generated by the* hmmalign* program is shown in Figure 2.

### 3.3. Determination of Consensus Epitopes

From the epitopes present in the 25 identified sites, 18 consensus epitopes generated using the Megalign program were obtained. These sites were distributed in the following manner: 6 for H1N1, 3 for H2N2, 3 for H3N2, and 6 for H5N1.

**Figure 3:**
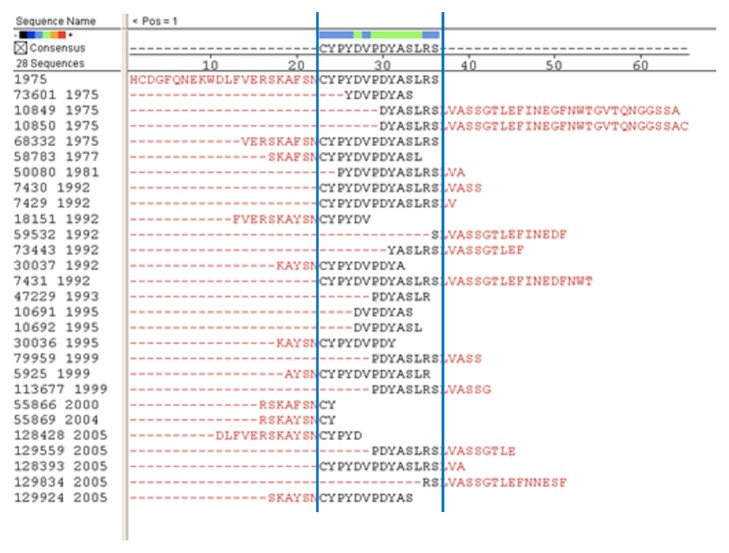
Example of the epitope alignment completed using the Megalign program. The depicted figure corresponds to the hemagglutinin of the H3N2 subtype. The blue lines show the consensus sequences in the different epitopes reported.

Upon completing the second alignment to determine the similitude percentages with strains that are currently circulating, it was observed that homology existed between 10 of the 18 consensus epitopes. In all cases, the similitude percentages were greater than 90% (Table 3 and Figure 3).

### 3.4. Three-Dimensional Arrangement of the Consensus Epitopes

With the objective of observing the level of exposure of the consensus epitopes in the three-dimensional protein structures, a mold was downloaded from PDB for each case with a similitude percentage ≥70% with the consensus sequence (Table 4).

**Figure 4:**
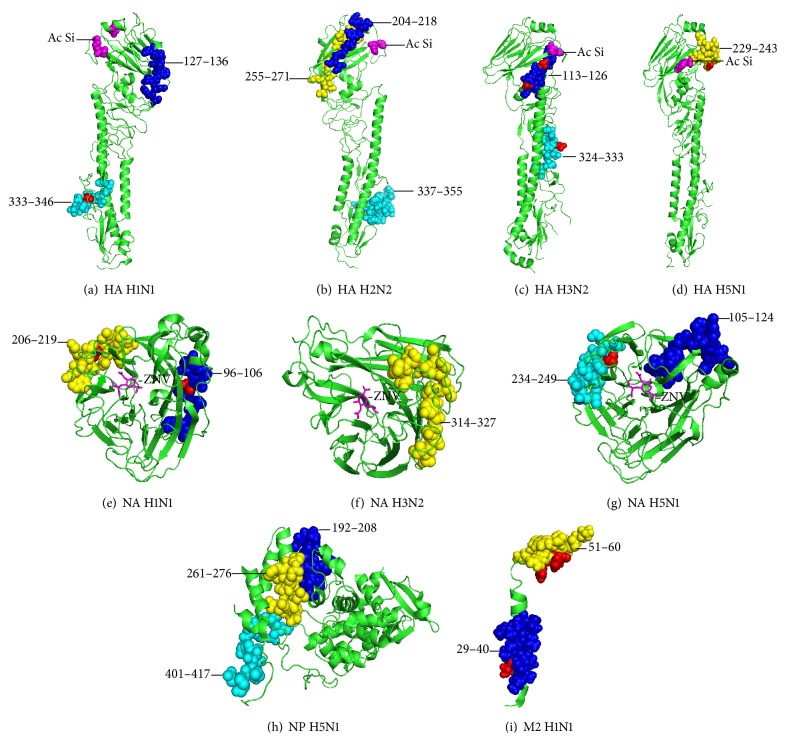
Three-dimensional localization of consensus epitopes. The protein structures are shown in green, and the conserved epitopes are shown as blue, yellow, and cyan spheres. The mutations within the consensus epitopes, compared to the current strains, are shown as red spheres. (a) [PDB: 2WR3] HA H1N1 in blue ^127^SVSSFERFEIFPK^136^; in cyan ^333^VTGLRNIPSIQSRGL^346^ (b) [PDB: 2WRD] HA H2N2 in blue ^204^LYQNVGTYVSVGTST^218^; in yellow ^255^FESTGNLIAPEYGFKIS^271^; in cyan ^337^IESRGLFGAIAGFIEGGWQ^355^ (c) [PDB: 2YP2] HA H3N2 in blue ^113^CYPYDVPDYASLRS^126^; in cyan ^324^YVKQNTLKLA^333^ (d) [PDB: 4BGW] HA H5N1 in yellow ^229^IATRSKVNGQSGRM^243^ (e) [PDB: 4B7J] NA H1N1 in blue ^96^GWAIYSKDNNS^106^; in yellow ^206^LKYNGIITETIKSW^219^ (f) [PDB: 4GZQ] NA H3N2 in yellow ^314^SSYVCSGLVGDTPR^327^ (g) [PDB: 4B7J] NA H5N1 in blue ^105^SHLECRTFFLTQGALLNDKH^124^; in cyan ^234^KIFKMEKGKVVKSVEL^249^ (h) NP H5N1 in blue ^192^ELIRMIKRGINDRNFWR^208^; in yellow ^261^RSALILRGSVAHKSCL^276^; in cyan ^401^ASAGQISVQPTFSVQRN^417 ^(i) [PDB: 2RLF] M2 H1N1 in blue ^29^AASIIGILHLIL^40^; in yellow ^51^ITRLFKHGLK^60^.

In the case of the HA protein, 5 of the 8 consensus epitopes were located in the globular zone, very close to the sialic acid-binding site, while the remaining epitopes were located in the stem region. For the NA protein, all of the defined epitopes were separate from the zanamivir-binding site. Finally, in the case of the M2 protein, consensus epitopes were identified in the membrane and transmembrane regions (Figure 4).

### 3.5. Phylogenetic Analysis

**Figure 5:**
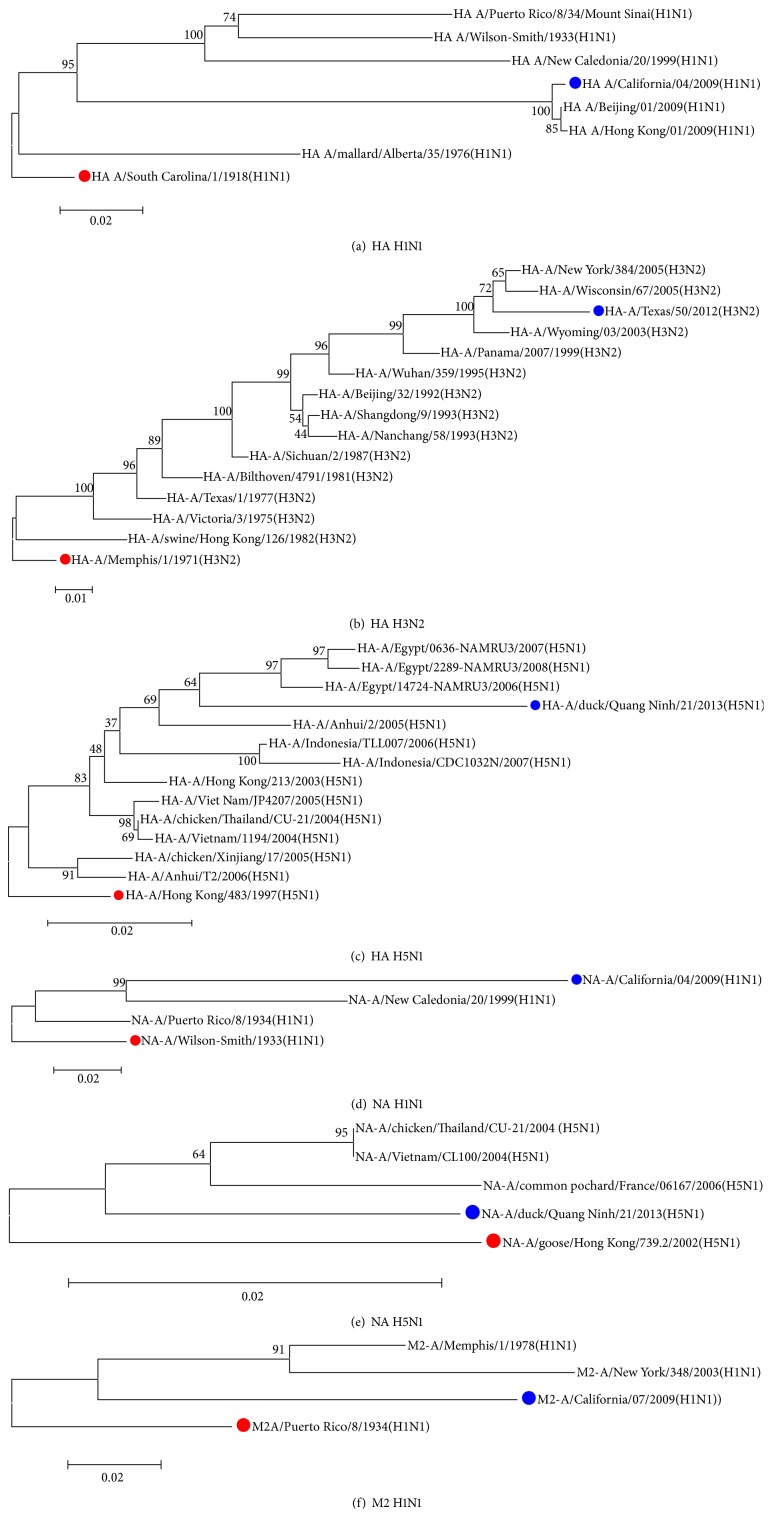
The red circle indicates the sequence in which the epitope was first identified. The blue circle shows the 2013-2014 season vaccine strain, in the case of H1N1 and H3N2, and the current strain, in the case of H5N1. The evolutionary history was inferred using the Neighbor-Joining method. The following branch shows the replication percentages with which the taxa associated in the bootstrap test. The trees are drawn to scale, with the longest of the branches in the same units of evolutionary distance used to infer the phylogenetic tree. The evolutionary distance was calculated using the Poisson method and is presented in substitution site units. The analysis was completed using the MEGA 6 program. Shown are (a) the optimum tree with a sum of branches of 0.43300672; (b) the optimum tree with a sum of branches of 0.30107033; (c) the optimum tree with a sum of branches of 0.20974655; (d) the optimum tree with a sum of branches of 0.29223355; (e) the optimum tree with a sum of branches of 0.07724998; (f) the optimum tree with a sum of branches of 0.28312403.

Phylogenetic analysis revealed the evolutionary distance between the sequence in which each epitope was identified for the first time and the sequence of either the 2013-2014 season vaccine strain (in the case of H1N1 and H3N2) or a strain reported in 2013 (in the case of H5N1). The mutation rates for nucleotide substitutions per site per year were 1.2 × 10^−3^ for HA H1N1, 3.5 × 10^−3^ for HA H3N2, 3.4 × 10^−3^ for HA H5N1, 1.6 × 10^−3^ for NA H1N1, 1.1 × 10^−4^ for NA H5N1, and 7.8 × 10^−4^ for M2 H1N1. For the HA H2N2, NA H2N2, and NP H5N1 proteins, a phylogenetic tree was not completed because the isolate sequences were sourced from 3 or fewer strains (Figure 5).

### 3.6. Geographic Distribution of Consensus Epitopes

**Figure 6:**
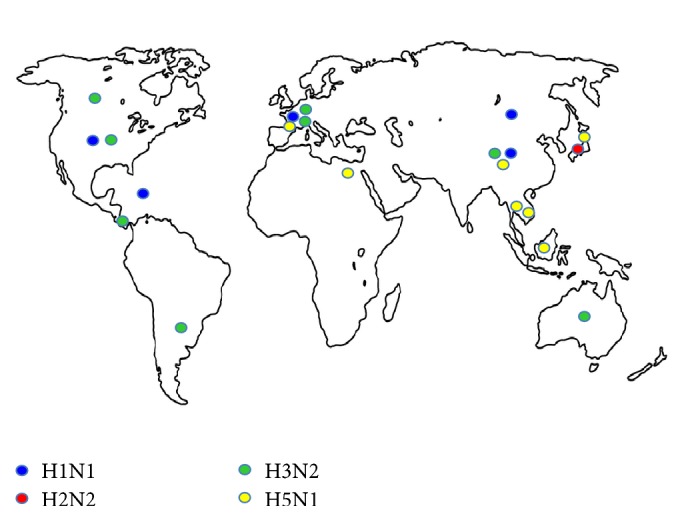
Geographic distribution of consensus epitopes. The countries in which the isolates containing the consensus epitopes defined in this study have been obtained are shown on the map.

The circulation analysis revealed that the epitopes defined in this study have been present in isolates obtained from 5 continents: the H1N1 epitopes have been identified in China, Mongolia, the USA, France, and Puerto Rico; the H2N2 epitopes have only been identified in Japan; the H3N2 epitopes were isolated in Panama, Argentina, Canada, the USA, China, Holland, France, and Australia; and, finally, the H5N1 epitopes were observed in Indonesia, Thailand, Vietnam, China, Japan, France, and Egypt (Figure 6).

## 4. Discussion

While the interest in the scientific community is focused on the production of an influenza vaccine that provides cross-protection against multiple subtypes [23], thus far, the vaccine must still be modified each year [20].

This necessity emphasizes the importance of identifying IAV sequence regions that remain constant and that, moreover, have the capacity to induce immunological responses from T and B cells. Therefore, this study completed an* in silico* analysis using previously reported epitopes and those for which evidence indicates they are capable of inducing an immunological response.

As was to be expected, given that H1N1 was the first IAV subtype that was recognized and has been circulating in the world since 1918, of the epitopes used in this study, those corresponding to H1N1 represented 44.8%, while those corresponding to H2N2 only represented 3%. This difference could, in part, be due to the fact that H2N2 only circulated after the 1957 pandemic up until 1968, when it was displaced by the H3N2 subtype [44, 45].

However, for all of the subtypes, with the exception of H2N2, the protein in which the greatest number of epitopes has been described is HA, with the following percentages: H1N1 50.7%, H3N2 52.2%, and H5N1 67.7%. This finding is similar to observations made by Bulimo and Cols in 2012 [46]. Interestingly, M2 is the protein for which fewer epitopes have been described, even though the M2 protein has been one of the main targets in studies focusing on vaccine development in the last few years [47–50].

It is important to note that although the H5N1 subtype has only been detected sporadically in humans, it is generally associated with cases of zoonosis in individuals who have very close contact with poultry or undomesticated birds as a result of work activities or hunting, respectively [51]. The epitopes reported for the H5N1 subtype represent 17.6% of the total, which tells us that it is worthy of study, as its high pathogenicity has been widely observed in both birds [52, 53] and humans [54, 55] since the first cases noted in 2003 [56]. Moreover, many authors believe that H5N1 could be the next strain capable of crossing the species barrier and obtaining the capacity of being transmitted from human to human [57]. Thus, it is incredibly important to be prepared for the eventual introduction of this subtype with studies such as this one that determine the consistent recognition zones that can be used as targets in epitope vaccination techniques. With the current methodology for producing influenza vaccines using embryonated eggs, an emergency with a new strain would require 6 to 8 months to create a vaccine [58], as occurred in 2009.

Studying the highly recognized zones or those zones with the greatest numbers of epitopes within the IAV proteins over the years is related to site conservation; their remaining constant makes it possible to identify the strains that will circulate in the coming years. Some of the consensus epitopes defined in this study are so constant that they have circulated for 91 years; these epitopes have been described in various studies conducted using sequences belonging to the 1918 strain and reported from 1983 (by Hackett and Cols) until 2012 (by Vergara and Cols). In the case of H2N2, there are epitopes that have been maintained for 44 years and have been described in studies completed from 1983 (by Lamb and Cols) until 2009 (by Babon and Cols). This study is the first to consider epitopes reported in strains that have circulated since 1918 to determine their conservation over such a long period of time.

It is not unusual that, for some IAV proteins, almost their entire sequence can be considered an epitope. Nevertheless, not all of these epitopes have the capacity to produce a protective response by inducing neutralizing antibodies, a fact that has been well verified in studies such as Gelder et al. in 1995 [37] and in 1998 [59], Alexander et al. in 2010 [43], and Rhee et al. in 2012 [40].

Of the 3,016 epitopes analyzed in this study, 18 consensus epitopes were identified, according to what was reported in the IEDB. Twelve of the epitopes were recognized exclusively by T lymphocytes [28, 31–35, 39, 43], while 6 represent overlapping antigens that are recognized by both B and T lymphocytes [29, 30, 32, 36–38, 40–42] (Table 3).

These epitopes were identified thanks to the fact that they have sequences of different sizes for the same site; thus, it was possible to determine the consensus sequence, that is, to eliminate the extremes from the reported sequences to determine, in an* in silico* manner, the minimum sequence that could be recognized by the immune system (Figure 3).

Another important aspect to take into consideration is how accessible or exposed these regions are for recognition by the immune system. In the case of the HA membrane protein, the majority of the consensus epitopes identified were located in the globular region (HA1); thus, we can infer that this region in general and, more specifically, the region close to the sialic acid-binding site show a greater degree of conservation than the stem region. These findings are different than those reported by Bulimo et al. in Kenya in 2012 [46] in a study that was completed during the 2007-2008 season; however, they support those findings reported by Iba et al. [60] in a study completed with the H3N2 strain with globular region epitopes with greater antigenic overlap than those found in the stem region. These findings emphasize the importance of conserving the cellular recognition region of the virus.

In the case of the NA protein, the consensus epitopes were identified far from the zanamivir-binding site, which would be expected, as this site is the site of greater selective pressure, resulting in a tendency for more modification. These findings coincide with those reported for the H1N1 subtype by Moscona in 2009 [61] and by Boivin in 2013 [62] and also for the H3N2 subtype by Tamura et al. in 2013 [63].

For the M2 protein, consensus epitopes were detected in the membrane and transmembrane regions, even though this is the Amantadine-binding site. Although resistance to ion channel blockers is less common for the M2 protein [64] than the resistance caused by neuraminidase (NA protein) inhibitors, it is hoped that the transmembrane region would have greater variability.

Another point to consider when searching for constant sites is the mutation rate for IAV proteins, which, contrary to what was expected, are 3-fold lower for the HA proteins of the H1N1 subtype than for HA proteins of the H3 and H5 subtypes. Similarly, the mutation rates are also 3-fold lower than that reported by Klein and colleagues [65] in 2014. This difference could occur because the periods of time evaluated were much shorter in those 3 cases, 41, 16, and 4 years, than the 91 years between the strains evaluated in this study. Another interesting fact is that the Klein study simply analyzed strains that circulated between 2009 and 2013 (from the pandemic), compared to our study, which analyzed strains from 1918 to 2009 (before the pandemic). Therefore, the mutation rate for the H1N1 subtype has apparently increased since 2009. This observation is reinforced if we consider that, between 1918 and 2009, the H1N1 subtype mutated due to genetic drift (i.e., only considering seasonal strains); yet, starting from the 2009 pandemic strain, modifications in its genome are the sum of both antigenic drift and antigenic shift (seasonal and pandemic strains). The same is true for the NA and NP proteins of the H1N1 subtype when comparing the findings from this study and those from Klein.

Finally, one aspect to explore is the reach of the vaccination or diagnostic strategies that could develop from these epitopes, as, currently, seasonal influenza vaccine design is based on strain circulation [66], independently for the northern [67] and southern hemispheres [68]. This fact increases the relevance of the consensus epitopes defined in this study, as, with the exception of the H2N2 subtype, all of the other subtypes have circulated on at least 3 continents, increasing their potential for use as therapeutic or diagnostic tools.

## 5. Conclusion

In conclusion, this study was able to identify 18 epitopes present in the HA, NA, NP, and M2 proteins of IAV that are, in accordance with previous studies, able to induce an immune response via T and B cells. These epitopes have remained constant for up to 91 years and have circulated on various continents. Nevertheless, because the analysis was completed in an* in silico* manner, it is necessary to demonstrate the potential of these findings experimentally in future studies.

## Figures and Tables

**Table 1:** Distribution of epitopes and sequences obtained for each subtype.

	Viral strains				
		H1N1	H2N2	H3N2	H5N1
		*n* (%)	*n* (%)	*n* (%)	*n* (%)
	HA				
Proteins	E	686 (50.7)	43 (47.3)	525 (52.2)	384 (67.8)
	S	8416 (55.7)	175 (42.5)	7313 (60.0)	3024 (45.9)
	NA				
	E	283 (20.9)	0 (0)	195 (19.4)	126 (22.2)
	S	4462 (29.6)	129 (31.3)	2909 (23.9)	1981 (30.0)
	NP				
	E	328 (24.3)	47 (51.6)	252 (25.0)	45 (7.9)
	S	1225 (8.1)	78 (18.9)	1096 (9.0)	1019 (15.5)
	M2				
	E	55 (4.1)	1 (1.1)	34 (3.4)	12 (2.1)
	S	999 (6.6)	30 (7.3)	868 (7.1)	570 (8.6)

Note: S = sequences and E = epitopes.

**Table 2:** Sites with the greatest frequency of epitopes.

Subtype	Protein	Site
H1N1	HA	123–140; 205–219; 330–352
	NA	93–109; 204–222
	NP	414–427
	M2	25–43; 48–62

H2N2	HA	200–220; 251–274; 332–356

H3N2	HA	112–131; 322–336;
	NA	143–160; 311–331
	NP	52–69; 363–378

H5N1	HA	174–195; 227–246; 247–267
	NA	103–121; 231–252
	NP	190–211; 257–278; 400–418

**Table 3:** Consensus epitopes.

Subtype	Protein	Epitope	Sequence	T or B cell	Reference
H1N1	HA	1	^127^SVSSFERFEIFPK^136^	T	[28]
		2	^333^VTGLRNIPSIQSRGL^346^	T y B	[29, 30]
	NA	3	^96^GWAIYSKDNNS^106^	T	[31]
		4	^206^LKYNGIITETIKSW^219^	T	[32]
	M2	5	^29^AASIIGILHLIL^40^	T	[33]
		6	^51^ITRLFKHGLK^60^	T	[32]

H2N2	HA	7	^204^LYQNVGTYVSVGTST^218^	T	[34]
		8	^255^FESTGNLIAPEYGFKIS^271^	T	[28]
		9	^337^IESRGLFGAIAGFIEGGWQ^355^	T	[35]

H3N2	HA	10	^113^CYPYDVPDYASLRS^126^	T y B	[32, 36]
		11	^324^YVKQNTLKLA^333^	T y B	[37, 38]
	NA	12	^314^SSYVCSGLVGDTPR^327^	T	[39]

H5N1	HA	13	^229^IATRSKVNGQSGRM^243^	T y B	[32, 40]
	NA	14	^105^SHLECRTFFLTQGALLNDKH^124^	T y B	[41, 42]
		15	^234^KIFKMEKGKVVKSVEL^249^	T y B	[32, 41]
	NP	16	^192^ELIRMIKRGINDRNFWR^208^	T	[43]
		17	^261^RSALILRGSVAHKSCL^276^	T	[39]
		18	^401^ASAGQISVQPTFSVQRN^417^	T	[39]

**Table 4:** PDB accession codes.

Subtype	Protein	PDB codes
H1N1	HA	2WR3
	NA	4B7J
	M2	2RLF

H2N2	HA	2WRD

H3N2	HA	2YP2
	NA	4GZQ

H5N1	HA	4BGW
	NA	4B7J
	NP	3TC6
